# Prevalence, subtypes, and associated factors of dementia in Taiwan: a nationwide survey of 11,298 community‐dwelling adults aged 65 years or older

**DOI:** 10.1002/alz70860_102326

**Published:** 2025-12-23

**Authors:** Ke‐Zong Michelle Ma, Chih‐Cheng Hsu

**Affiliations:** ^1^ National Center for Geriatrics and Welfare Research, National Health Research Institutes, Miaoli County, Taiwan

## Abstract

**Background:**

The rapid growth of the older adult population requires a greater epidemiological characterization of dementia, which is crucial for estimating the disease burden and planning healthcare. This comprehensive study aimed to assess the prevalence, risk factors, and subtypes of dementia in Taiwan.

**Method:**

Using the stratified multi‐stage cluster sampling method, we conducted a two‐phase screening design to ascertain dementia among adults aged 65 years and older from 22 cities/counties across the country. In the first stage, well‐trained interviewers conducted face‐to‐face home interviews to collect information about the older adults’ demographic profiles and dementia‐related scales, including Ascertain Dementia 8 questionnaire and Mini‐Mental State Examination. Based on the results derived from the first‐stage survey, 2,403 people suspected of cognitive impairment were identified to receive face‐to‐face home interviews by well‐trained physicians for clinical ascertainment of dementia through the diagnosis principles of NINCDS‐ADRDA. Risk factors for dementia were examined using multivariable‐adjusted analyses.

**Result:**

The weighted prevalence of dementia in Taiwan was 7.99%. Dementia prevalence among women (9.36%) was higher than men (6.35%). Dementia was more prevalent among illiterate individuals (15.93%). Dementia prevalence increased with age, from 2.40% of those aged 65–69 years to 29.45% of those aged 90 and older. The most common subtype was Alzheimer's disease (56.88%), followed by vascular dementia (22.91%), Parkinson's disease dementia (7.12%), dementia with Lewy body (2.63%), and frontotemporal dementia (0.99%). The remaining 69 patients had other diagnoses, including combinations of dementia disorders other than combined Alzheimer's and vascular pathology. Risk factors for dementia included old age (odds ratio ranging from 1.81 [95% CI: 1.36‐2.41] to 7.00 [5.28‐9.28]), female sex (odds ratio: 1.34 [1.11‐1.60]), fewer years of education (odds ratio: 1.45 [1.22‐1.59]), low income (odds ratio: 2.06 [1.33‐3.20]), cerebrovascular disease (odds ratio: 3.61 [2.74‐4.71]), and Geriatric Depression Scale‐5 (GDS‐5) score≧2 (odds ratio: 3.72 [3.10‐4.47]).

**Conclusion:**

Consistent with common findings from other parts of the world, a high rate of dementia was associated with female sex, older age, lower education, and depression. Alzheimer's disease was the most common cause. The findings of this study can help prioritize dementia research, clinical services, and care resources in Taiwan.

